# Factors associated with behavioral engagement and learning satisfaction in YouTube-based informal English learning among Taiwanese undergraduate students

**DOI:** 10.1371/journal.pone.0357471

**Published:** 2026-08-28

**Authors:** Jung-Wen Hsia, Aihua Tseng

**Affiliations:** 1 Department of Counseling Psychology and Human Resource Development, National Chi Nan University, Nantou County, Taiwan; 2 Center for Continuing Education, National Tsing Hua University, Hsinchu City, Taiwan; Universiti Sains Malaysia, MALAYSIA

## Abstract

As learners increasingly use open online resources for informal language learning, YouTube has become an important setting for self-directed English learning. However, less is known about how learners’ motivation, confidence in using YouTube for learning, and perceptions of content quality are associated with behavioral engagement and learning satisfaction in this context. This study examined the associations of perceived usefulness, perceived enjoyment, YouTube learning self-efficacy, and content quality with behavioral engagement and learning satisfaction in YouTube-based informal English learning (YIEL). It also examined whether behavioral engagement statistically mediated the associations of these four constructs with learning satisfaction. Data were collected through a cross-sectional survey of 393 undergraduate students in Taiwan and analyzed using partial least squares structural equation modeling (PLS-SEM). All hypothesized associations were supported, and the model explained 47.6% of the variance in behavioral engagement and 64.7% of the variance in learning satisfaction. Behavioral engagement also statistically mediated the associations between these four constructs and learning satisfaction. The findings indicate that perceived usefulness, perceived enjoyment, YouTube learning self-efficacy, and content quality were associated with both behavioral engagement and learning satisfaction, and that behavioral engagement was also associated with learning satisfaction. Together, these findings extend current understanding of YIEL by highlighting behavioral engagement both as an outcome associated with these four focal constructs and as a statistical mediator of their associations with learning satisfaction.

## Introduction

Online, distance, and social-media-supported learning have received sustained attention in educational research [1], and their role has become more prominent in recent years. The COVID-19 pandemic further accelerated this trend as institutions expanded digital learning environments [2]. Beyond formal settings, learners have increasingly used open online resources to support informal learning [3,4]. YouTube is particularly relevant because it is widely used for self-directed and informal learning [5,6].

Since its launch in 2005, YouTube has gradually become an important platform for video-based and informal language learning [3,7,8]. Unlike course-based platforms, learning materials on YouTube are not usually organized into a predetermined curriculum or formal instructional sequence**.** Learners therefore encounter and select learning materials in a more open and less institutionally guided environment. Its multimodal format supports flexible and self-paced learning. However, because YouTube is open and largely user-generated, learning materials may vary in relevance, reliability, and the extent to which they support learning [3,9]. Learners therefore carry much of the responsibility for judging whether a video is useful, credible, and suitable for their own English learning needs.

Although YouTube has received increasing attention as a learning tool, prior research has often focused on classroom-based or teacher-guided contexts [8,10]. These studies provide useful evidence on YouTube-supported learning. However, they may not fully explain how learners evaluate, select, and sustain engagement with English learning videos in an open environment. This environment is shaped by algorithmic recommendations and uneven content quality. In YIEL, learners tend to make these decisions more independently, while their exposure to learning materials may also be shaped by search functions, subscriptions, social sharing, and algorithmic recommendations. The joint associations of motivational factors, YouTube learning self-efficacy, and content quality with behavioral engagement and learning satisfaction therefore require further clarification.

This study extends prior work in two ways. First, it examines motivational factors, YouTube learning self-efficacy, and content quality within the same framework. Second, it gives greater attention to behavioral engagement by examining it both as an outcome and as a statistical mediator of the associations between the four focal constructs and learning satisfaction.

Accordingly, this study addresses the following two research questions:

RQ1. How are perceived usefulness, perceived enjoyment, YouTube learning self-efficacy, and content quality associated with behavioral engagement in YIEL contexts?

RQ2. In YIEL contexts, how are perceived usefulness, perceived enjoyment, YouTube learning self-efficacy, and content quality directly associated with learning satisfaction, and what specific indirect associations involving behavioral engagement are observed?

### Literature review and hypotheses development

#### YouTube-based informal English learning.

Prior research on YIEL-related topics can be grouped into three main areas. One early line of work examined linguistic aspects of informal language learning, showing that YouTube can serve as a source of English exposure and language use beyond formal instruction [11]. A second line of research has examined learners’ motivational, affective, and self-regulatory processes, highlighting the role of learner agency in YouTube-based English learning [5,6]. A third line of research has examined learners’ experiences with YouTube as a learning environment, with particular attention to psychological perceptions, platform-related evaluations, content quality, engagement, and learning outcomes [3,4,12]. Taken together, these studies suggest that YIEL involves not only language exposure, but also learners’ motivation, agency, platform-related perceptions, and evaluations of content quality.

However, these factors have often been examined separately. Given the learner-directed and content-diverse nature of YIEL, it is important to consider motivational factors, YouTube learning self-efficacy, and content quality together when examining behavioral engagement and learning satisfaction. The following sections review these three main dimensions in turn: learner motivation, learner self-efficacy, and content quality.

#### Learner motivation.

In this study, perceived usefulness and perceived enjoyment are treated as two complementary aspects of learner motivation. Perceived usefulness can be understood as a more extrinsic, goal-oriented aspect of motivation, whereas perceived enjoyment reflects intrinsic motivation derived from the learning activity itself [13,14]. Prior studies have shown that learner motivation is closely related to engagement-related outcomes in technology-enhanced learning contexts [15,16]. Drawing on technology acceptance and intrinsic motivation perspectives, perceived usefulness and perceived enjoyment are discussed below as two motivational factors in YIEL.

#### Technology acceptance model and perceived usefulness.

Drawing on the Technology Acceptance Model [17], perceived usefulness is used in this study to describe learners’ beliefs about whether YouTube can support their English learning goals. Although the Technology Acceptance Model also includes perceived ease of use, this construct may be less central in the present context. In prior research on YouTube-based procedural learning, perceived ease of use was not significantly associated with perceived usefulness or behavioral intention, whereas perceived usefulness showed a significant association with behavioral intention [18]. For this reason, the present study focuses on perceived usefulness as a key motivational factor.

Studies of technology-enhanced learning have generally shown that learners who perceive a platform as useful tend to report more favorable engagement-related and satisfaction-related outcomes [19,20]. Similar patterns have also been found in language learning contexts [21,22]. However, YIEL differs from many of these settings because learners engage with open and user-generated materials with limited formal support. Their exposure to available resources may also be shaped by platform mechanisms such as search results and algorithmic recommendations [3,9]**.** In this context, perceived usefulness may be especially important because learners need to judge whether the videos they encounter are worth continued attention and effort.

In this context, perceived usefulness reflects learners’ broader belief that YIEL can support their English learning goals through available platform resources. Therefore, learners’ perceived usefulness of YouTube for English learning may be associated with stronger behavioral engagement and greater learning satisfaction. Accordingly, this study proposes the following hypotheses:

H1. Perceived usefulness is positively associated with behavioral engagement.

H2. Perceived usefulness is positively associated with learning satisfaction.

#### Intrinsic motivation theory and perceived enjoyment.

According to Intrinsic Motivation Theory [14], learners may participate in an activity because the activity itself is interesting, enjoyable, or satisfying. In technology-mediated learning contexts, perceived enjoyment refers to learners’ affective response to using a system because the experience itself is enjoyable. It reflects the intrinsic motivational side of technology use. Previous studies have reported that perceived enjoyment is associated with favorable attitudes and continued use in digital and hedonic systems [13,23].

Prior empirical studies have also found that perceived enjoyment is positively associated with behavioral engagement and learning satisfaction in technology-supported and language learning environments [20,21,24]. However, the associations involving perceived enjoyment may differ between YIEL and more structured online learning settings. In the less structured YouTube environment, English learning content may include informal presentation styles, multimedia elements, and entertainment-oriented features [7,12]. These elements may be associated with a less burdensome learning experience and stronger behavioral engagement in the absence of formal instructional guidance.

In this learner-directed context, the affective experience of using YouTube for English learning may also be closely related to learners’ overall evaluation of the learning process. Accordingly, enjoyable learning experiences may be associated with stronger behavioral engagement and more positive evaluations of YouTube-based English learning. Therefore, this study proposes the following hypotheses:

H3. Perceived enjoyment is positively associated with behavioral engagement.

H4. Perceived enjoyment is positively associated with learning satisfaction.

#### YouTube learning self-efficacy.

YouTube learning self-efficacy represents the learner self-efficacy dimension in the proposed model. Building on Social Cognitive Theory [25,26], it is conceptualized in this study as learners’ confidence in performing platform-specific English learning tasks on YouTube. These tasks include searching for relevant videos, navigating the platform, selecting and using videos for learning, and exploring new learning resources. This conceptualization adapts the broader notion of online learning self-efficacy [27] to the specific learning demands of YIEL. It is also consistent with Bandura’s view that self-efficacy concerns individuals’ beliefs in their ability to perform actions required to achieve specific goals [25,26]. Thus, YouTube learning self-efficacy refers to confidence in managing English learning tasks on YouTube, rather than general confidence in using online learning tools.

Because YouTube learning self-efficacy has received less direct attention as a distinct construct in prior YIEL research, related studies on online learning self-efficacy offer a useful reference point. Prior research has reported positive associations of online learning self-efficacy with behavioral engagement in online English courses [28] and with learning satisfaction in blended or platform-based learning environments [29,30]. However, these findings may not fully explain YIEL, where learning occurs without a structured course sequence and learners must rely more on their own decisions when using available videos.

YouTube learning self-efficacy is particularly relevant in YIEL because learners must manage learning tasks without a structured course sequence. Stronger YouTube learning self-efficacy may therefore be associated with greater behavioral engagement and more positive evaluations of the learning experience. Thus, the present study proposes the following hypotheses:

H5. YouTube learning self-efficacy is positively associated with behavioral engagement.

H6. YouTube learning self-efficacy is positively associated with learning satisfaction.

#### Content quality.

Content quality represents the content-related dimension in the proposed model. Drawing on the Information Systems Success Model [31,32], this study contextualizes information quality as content quality in YIEL. Since learning information on YouTube is primarily embedded in English learning videos, content quality refers to learners’ evaluations of whether these videos are current, relevant, reliable, and accurate. Because YouTube relies heavily on user-generated videos, such evaluations are especially important in YIEL. Learners must judge the suitability and credibility of available learning resources with limited formal instructional guidance.

Previous research has shown that content-related factors, such as course content, are associated with learner engagement and satisfaction in structured online and blended learning environments [33]. Consistent with the Information Systems Success Model [31,32], favorable evaluations of information quality are theoretically expected to be associated with use-related outcomes and user satisfaction. In YIEL, learners who perceive English learning videos as current, relevant, reliable, and accurate may also regard these videos as more worthwhile learning resources. Such favorable evaluations are expected to be positively associated with behavioral engagement, as reflected in the time and effort learners devote to learning activities. Perceptions that the available content meets learners’ informational needs and learning expectations are also expected to be positively associated with learning satisfaction.

In algorithm-mediated YIEL, learners may locate English-learning videos through YouTube’s search features [9], while YouTube’s algorithmic recommendation system may partly shape the videos they encounter [34]. Consequently, learners evaluate the quality of a pool of videos shaped jointly by their own selection practices and algorithmic platform curation. Because algorithmic curation helps determine which videos learners encounter and subsequently evaluate, it may partially substitute for the deliberate comparison and screening that learners would otherwise undertake during content selection. This content-selection context differs from more structured learning environments, where instructors or institutions typically preselect and organize materials according to instructional objectives. Whether the expected positive associations of perceived content quality with behavioral engagement and learning satisfaction also hold in this mixed content-selection context therefore requires empirical examination. Accordingly, the following hypotheses are proposed:

H7. Content quality is positively associated with behavioral engagement.

H8. Content quality is positively associated with learning satisfaction.

#### Behavioral engagement.

Behavioral engagement refers to the behavioral side of learner engagement, including participation, effort, persistence, and attention in learning activities [35]. In YIEL, this construct is reflected in learners’ active involvement with English learning videos, such as sustained attention, careful watching and listening, and effortful engagement with learning materials. This focus is appropriate because YIEL depends heavily on learners’ own participation rather than teacher-assigned activities.

Although behavioral engagement was originally conceptualized mainly in structured classroom settings [35,36], its core elements, including effort, attention, and persistence, are also relevant in self-directed learning environments. In YIEL contexts, behavioral engagement reflects learners’ active involvement in selecting and using learning content without formal instructional guidance. This contextual understanding supports the use of behavioral engagement in YIEL, while recognizing that engagement in this context may differ from engagement in formal classroom settings.

Earlier studies in online learning have reported positive associations between behavioral engagement, learner satisfaction, and academic outcomes [37,38]. In this learner-directed context, learning satisfaction may be related to whether learners can sustain attention, effort, and time investment during the learning process. Such active involvement may therefore be associated with learners’ evaluations of their YouTube-based English learning experience. Accordingly, the present study examines the association between behavioral engagement and learning satisfaction in YIEL contexts and proposes the following hypothesis:

H9. Behavioral engagement is positively associated with learning satisfaction.

In addition to its direct association with learning satisfaction, behavioral engagement is examined as a statistical mediator of the associations of perceived usefulness, perceived enjoyment, YouTube learning self-efficacy, and content quality with learning satisfaction. In YIEL, learners’ positive beliefs and evaluations may be associated with satisfaction both directly and indirectly, with behavioral engagement involved in the indirect associations. This possibility is examined by testing whether these four constructs have indirect associations with learning satisfaction involving behavioral engagement. Prior studies in technology-mediated learning have reported that engagement statistically mediated associations between learner- or learning-environment-related factors and online learning satisfaction [37,38]. Accordingly, the following mediation hypotheses are proposed:

H10a. Behavioral engagement statistically mediates the association between perceived usefulness and learning satisfaction.

H10b. Behavioral engagement statistically mediates the association between perceived enjoyment and learning satisfaction.

H10c. Behavioral engagement statistically mediates the association between YouTube learning self-efficacy and learning satisfaction.

H10d. Behavioral engagement statistically mediates the association between content quality and learning satisfaction.

#### Research model.

**Fig 1:**
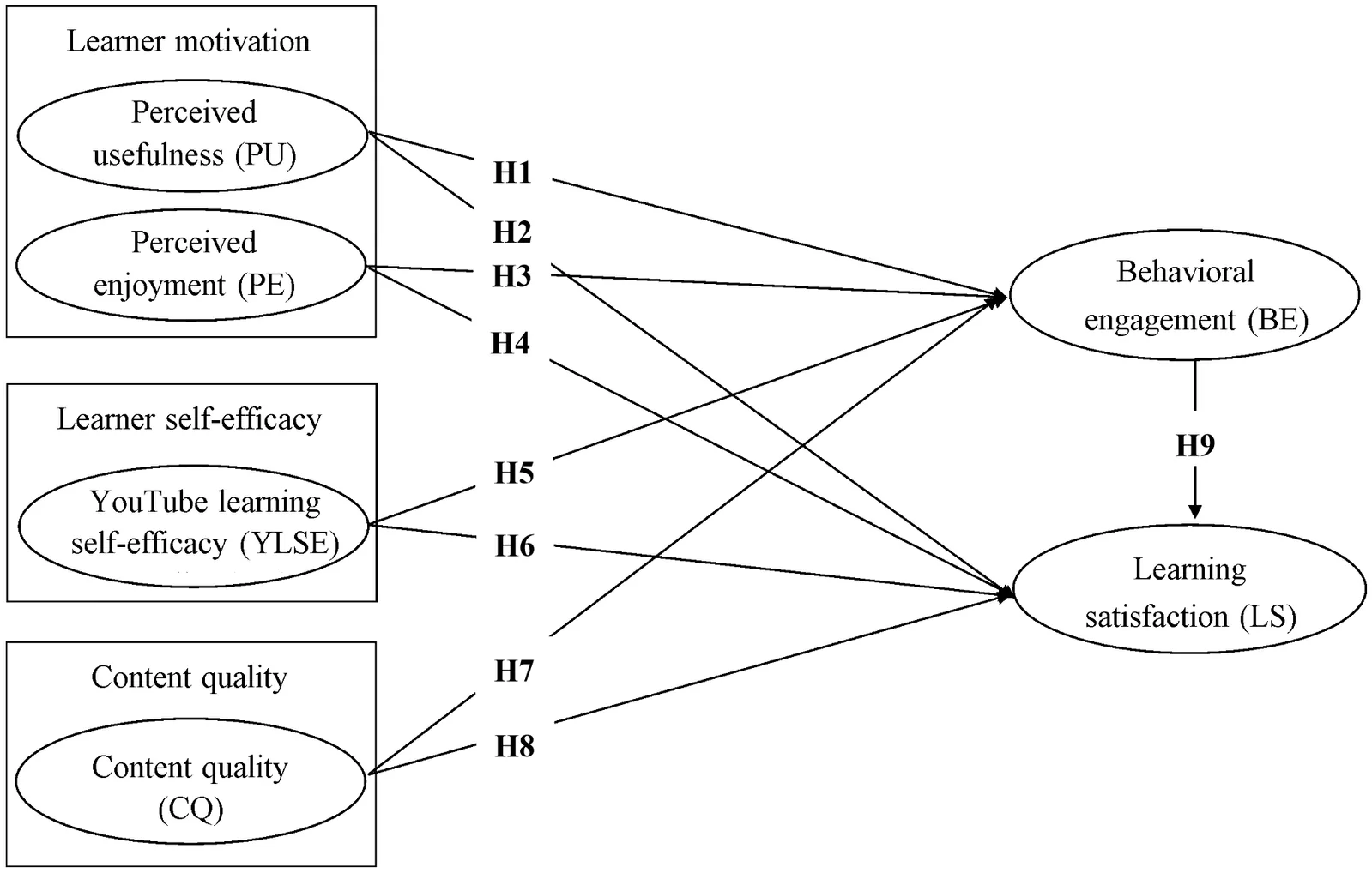
Research model.

Building on the theoretical frameworks and empirical evidence reviewed above, this study proposes an integrated research model for YIEL. The model specifies associations of perceived usefulness, perceived enjoyment, YouTube learning self-efficacy, and content quality with behavioral engagement and learning satisfaction. Behavioral engagement is specified as directly associated with learning satisfaction. It is also examined as a statistical mediator between these four constructs and learning satisfaction (Fig 1).

## Methods

### Measures

All constructs were measured using multi-item scales adapted from prior validated instruments. These scales have demonstrated acceptable reliability and validity in previous studies. Item wording was adapted where necessary to maintain semantic equivalence and relevance to YIEL. Theoretical constructs were defined and operationalized as shown in Table 1, and all constructs were modeled as reflective.

**Table 1:** Definitions of theoretical constructs.

Theoretical construct	Operational definition	Measurement source
Perceived Usefulness (PU)	The extent to which an individual believes that learning English through YouTube would support learning goals.	[17]
Perceived Enjoyment (PE)	The extent to which individuals find learning English via YouTube intrinsically enjoyable.	[39]
YouTube Learning Self-Efficacy (YLSE)	The extent to which learners feel confident in performing YouTube-based English learning tasks, including searching for relevant videos, navigating the platform, selecting and using videos for learning, and exploring new learning resources.	[18]
Content Quality (CQ)	The extent to which learners perceive that English learning videos on YouTube are timely, relevant, reliable, and accurate.	[40]
Behavioral Engagement (BE)	The extent of learners’ attention, effort, and persistence while engaging with English learning videos via YouTube.	[36]
Learning Satisfaction (LS)	The perceived degree of contentment with learners’ overall evaluative experience of learning English through YouTube.	[19]

Perceived usefulness was measured using four items adapted from [17]. Perceived enjoyment was assessed with three items adapted from [39]. YouTube learning self-efficacy was measured using four items adapted from prior YouTube self-efficacy scales [18]. Content quality was measured using four items adapted from [40]. Behavioral engagement was measured using four items adapted from [36]. Learning satisfaction was measured with three items adapted from [19]. All items were rated on a five-point Likert scale ranging from 1 (strongly disagree) to 5 (strongly agree). The questionnaire items are provided in S1 Appendix.

Additional explanation is provided below for YouTube learning self-efficacy and behavioral engagement because these two adapted scales required contextual clarification for use in YIEL.

YouTube learning self-efficacy was adapted from prior YouTube self-efficacy scales [18]. These scales assess users’ general abilities on the platform, such as navigating the interface, searching for content, and browsing videos. In this study, the items were revised to fit the context of YIEL. The focus was on learners’ confidence in performing learning-related tasks, such as searching for relevant videos, navigating the platform efficiently, selecting and using videos for learning, and exploring new learning resources on their own. For example, a general item such as “I feel confident navigating YouTube” was revised to “I feel confident navigating YouTube to locate relevant English learning videos efficiently.” These changes are consistent with the task-specific nature of self-efficacy [26] and reflect the learning demands of YIEL. Thus, YouTube learning self-efficacy refers to platform-specific self-efficacy for English learning rather than general YouTube use.

Behavioral engagement was measured using four items adapted from [36], originally developed for formal classroom settings. The items were revised to fit the context of YIEL and to capture learners’ active involvement in video-based learning, including attention, effort, and persistence. Although YIEL takes place outside formal classroom settings, these behavioral indicators may still appear when learners devote attention, invest effort, and maintain focus while engaging with English learning videos without teacher-assigned requirements. Thus, the adapted items focus on observable learning actions that may occur in less structured, technology-mediated learning settings.

As the target participants were Taiwanese undergraduate students, the questionnaire was administered in Traditional Chinese. A back-translation procedure was used to enhance conceptual consistency between the English and Chinese versions. The English items were first translated into Chinese by a bilingual researcher and then independently back-translated into English by another bilingual individual. Discrepancies were discussed and revised. The Chinese questionnaire was also reviewed by experts in educational research and questionnaire design to evaluate item clarity and cultural appropriateness, and minor wording adjustments were made based on their feedback.

To further ensure measurement consistency and a common response frame, participants received instructions before responding to the questionnaire items. They were asked to base their responses on their general experiences of using YouTube for informal English learning. For clarification, informal English learning was described as voluntarily watching English-related videos for self-directed learning purposes outside formal classroom settings. Participants were explicitly instructed to exclude any classroom-related or instructor-assigned use of the platform.

### Participants and data collection

Participants were undergraduate students enrolled in a public university in central Taiwan. Because English is taught as a foreign language (EFL) in Taiwan, this group was considered relevant for examining informal English learning through digital platforms such as YouTube.

Convenience sampling was employed, and participation was voluntary. Recruitment announcements were disseminated through a private, university-affiliated Facebook community. Interested individuals completed a paper-based questionnaire at the university library between October 1 and October 22, 2024.

Participant eligibility was assessed using a screening question that examined whether respondents had engaged in learner-initiated English learning via YouTube during the past three months. The analytical sample therefore included only those with such experience within that period.

The study protocol was approved by the Central Regional Research Ethics Committee, China Medical University, Taichung, Taiwan (Approval No. CRREC-113–075). Written informed consent was obtained from all participants, all of whom were aged 18 years or older. The signed consent forms were securely stored and accessible only to the research team. Questionnaire responses were collected anonymously and analyzed in aggregate form to ensure confidentiality.

Of the 396 questionnaires collected, three were excluded due to substantial missing data, leaving 393 valid responses for analysis. The final sample comprised 205 males (52.2%) and 188 females (47.8%). Sophomore students represented the largest group (*n* = 122, 31.0%), followed by juniors (*n* = 113, 28.8%). Participants came from four colleges, with the College of Science and Technology forming the largest group (*n* = 125, 31.8%), followed by Management, Education, and Humanities. Most participants reported one year or less (*n* = 165, 42.0%) or one to two years (*n* = 149, 37.9%) of prior YIEL experience. For average monthly YIEL frequency, the largest group reported using YouTube for informal English learning 4–6 times per month (*n* = 170, 43.3%). Detailed demographic and learning-related characteristics of the sample are summarized in Table 2.

**Table 2:** Demographic and learning-related characteristics of participants (*N* = 393).

Characteristic	Category	Frequency, *n*	Percentage, %
Gender	Male	205	52.2
	Female	188	47.8
Academic level	Freshman	89	22.6
	Sophomore	122	31.0
	Junior	113	28.8
	Senior	69	17.6
College	Science and Technology	125	31.8
	Management	109	27.7
	Education	95	24.2
	Humanities	64	16.3
Years of experience with YIEL	1 year or less	165	42.0
	1–2 years	149	37.9
	2–3 years	46	11.7
	More than 3 years	33	8.4
Average monthly frequency of YIEL	1–3 times	128	32.6
	4–6 times	170	43.3
	More than 6 times	95	24.2

Percentages may not total 100 due to rounding.

### Data analysis

Partial Least Squares Structural Equation Modeling (PLS-SEM) was used to address the research questions and evaluate the proposed model. The analysis was conducted using SmartPLS 4.0 [41].

Although the constructs in this study are theoretically established, the primary aim was to examine associations among multiple constructs within an integrated framework. The study was conducted in the context of YIEL, which remains relatively underexplored. The model integrates perspectives from the Technology Acceptance Model, Social Cognitive Theory, the Information Systems Success Model, and Intrinsic Motivation Theory. It also includes multiple latent constructs and simultaneous associations. For these reasons, PLS-SEM was considered appropriate for the present study. This approach is particularly suitable when the research emphasizes variance explanation and the estimation of associations among latent constructs rather than covariance-based model fit evaluation [42]. Nevertheless, covariance-based structural equation modeling (CB-SEM) may also be suitable for future confirmatory replications focused on evaluating overall model fit.

A statistical power analysis was conducted using G*Power 3 [43] to estimate the minimum required sample size for a linear multiple regression model. Assuming a medium effect size (*f*² = 0.15), a significance level of .05, statistical power of .80, and five predictors of the most complex endogenous construct, the minimum required sample size was 92. The final analytical sample of 393 exceeded this requirement.

To ensure an appropriate evaluation sequence, this study adopted the two-step modeling approach [44]. In the first step, the measurement model was assessed to examine construct reliability and validity. After establishing an acceptable measurement model, the structural model was subsequently analyzed to test the hypothesized associations among constructs. The statistical significance of the structural paths and indirect associations was evaluated using a bootstrapping procedure with 5,000 resamples.

## Results

### Measurement model

The measurement model was evaluated using Cronbach’s α, composite reliability (CR), convergent validity, and discriminant validity. In addition, common method bias (CMB) was assessed as a supplementary diagnostic concern.

Initially, Cronbach’s α and CR were calculated to assess internal consistency. As shown in Table 3, Cronbach’s α values ranged from 0.726 to 0.828, and CR values ranged from 0.828 to 0.886. All values exceeded the recommended threshold of 0.7 [45], indicating acceptable internal consistency across all constructs**.** Convergent validity was evaluated using the average variance extracted (AVE) and factor loadings. All AVE values (0.546–0.712) exceeded 0.5 [46], and all factor loadings (0.704–0.865) were above 0.7 [47], indicating adequate convergent validity across all constructs.

**Table 3:** Reliability and convergent validity.

Construct	Cronbach’s α	Composite Reliability	Items	Factor Loadings	AVE
Perceived Usefulness (PU)	0.828	0.886	PU1	0.814	0.660
			PU2	0.817	
			PU3	0.813	
			PU4	0.805	
Perceived Enjoyment (PE)	0.797	0.881	PE1	0.822	0.712
			PE2	0.865	
			PE3	0.843	
YouTube learning self-efficacy (YLSE)	0.773	0.854	YLSE1	0.771	0.594
			YLSE2	0.774	
			YLSE3	0.774	
			YLSE4	0.763	
Content Quality (CQ)	0.726	0.828	CQ1	0.704	0.546
			CQ2	0.775	
			CQ3	0.738	
			CQ4	0.738	
Behavioral Engagement (BE)	0.812	0.877	BE1	0.806	0.640
			BE2	0.821	
			BE3	0.779	
			BE4	0.793	
Learning Satisfaction (LS)	0.765	0.864	LS1	0.859	0.680
			LS2	0.819	
			LS3	0.795	

Discriminant validity was examined using three complementary approaches. First, as shown in Table 4, the square root of the AVE for each construct exceeded its correlations with other constructs, supporting the Fornell–Larcker criterion [46]. Second, all Heterotrait–Monotrait Ratio (HTMT) values were below the recommended threshold of 0.90, and the 95% bootstrapped confidence intervals did not include 1.0 [48]. Third, cross-loadings confirmed that each item loaded higher on its intended construct than on other constructs [48] (Table 5). These results support satisfactory discriminant validity.

**Table 4:** Discriminant validity.

	PU	PE	YLSE	CQ	BE	LS
PU	**0.812**	0.688	0.713	0.787	0.712	0.823
PE	0.559	**0.844**	0.623	0.746	0.655	0.760
YLSE	0.572	0.491	**0.771**	0.810	0.721	0.825
CQ	0.615	0.567	0.610	**0.739**	0.746	0.854
BE	0.583	0.529	0.575	0.581	**0.800**	0.884
LS	0.655	0.594	0.637	0.648	0.698	**0.825**

The diagonal contains the square roots of the AVE, displayed in bold, while the lower triangle shows the correlations between constructs and the upper triangle presents the HTMT values. Construct abbreviations are defined in Table 1.

**Table 5:** Factor structure matrix of loadings and cross-loadings.

Items	PU	PE	YLSE	CQ	BE	LS
PU1	**0.814**	0.476	0.450	0.477	0.466	0.511
PU2	**0.817**	0.473	0.476	0.513	0.469	0.558
PU3	**0.813**	0.453	0.449	0.495	0.485	0.538
PU4	**0.805**	0.415	0.483	0.512	0.473	0.521
PE1	0.471	**0.822**	0.393	0.460	0.410	0.490
PE2	0.487	**0.865**	0.457	0.524	0.495	0.501
PE3	0.458	**0.843**	0.390	0.449	0.429	0.515
YLSE1	0.477	0.393	**0.771**	0.473	0.505	0.518
YLSE2	0.428	0.374	**0.774**	0.467	0.389	0.512
YLSE3	0.447	0.405	**0.774**	0.520	0.444	0.455
YLSE4	0.405	0.339	**0.763**	0.419	0.426	0.474
CQ1	0.400	0.435	0.423	**0.704**	0.366	0.383
CQ2	0.476	0.405	0.461	**0.775**	0.474	0.558
CQ3	0.452	0.410	0.433	**0.738**	0.375	0.434
CQ4	0.481	0.433	0.480	**0.738**	0.481	0.512
BE1	0.468	0.435	0.456	0.470	**0.806**	0.569
BE2	0.457	0.401	0.514	0.467	**0.821**	0.599
BE3	0.487	0.387	0.442	0.445	**0.779**	0.513
BE4	0.456	0.468	0.424	0.477	**0.793**	0.549
LS1	0.563	0.528	0.553	0.571	0.601	**0.859**
LS2	0.527	0.477	0.553	0.530	0.583	**0.819**
LS3	0.531	0.463	0.467	0.500	0.541	**0.795**

Construct abbreviations are defined in Table 1.

To address potential concerns regarding CMB, both procedural and statistical steps were considered. Procedurally, participants were assured of anonymity and confidentiality, and responses were analyzed in aggregate form. Statistically, Harman’s single-factor test showed that the first factor accounted for 41.9% of the total variance, below the 50% threshold [49]. In addition, inner VIFs were examined as a supplementary diagnostic check. As reported in the structural model assessment, all inner VIF values were below the commonly accepted threshold of 5 [50], suggesting that serious collinearity-related concerns were unlikely. Taken together, these results suggest that common method bias is unlikely to have substantially biased the findings, although it cannot be entirely ruled out.

In summary, the measurement model showed satisfactory internal consistency, convergent validity, and discriminant validity across all constructs. These results support the reliability and validity of the measurement model and provide an acceptable basis for the subsequent structural model analysis.

### Structural model

As noted above, inner variance inflation factors (VIFs) were examined to assess multicollinearity among the predictor constructs for each endogenous construct. The results indicated no major multicollinearity concerns, as all VIF values were below the commonly accepted threshold of 5 [50], as shown in Table 6.

**Table 6:** Inner VIF values for the structural model.

	PU	PE	CQ	YLSE	BE	LS
PU					1.931	2.031
PE					1.677	1.732
YLSE					1.799	1.909
CQ					2.064	2.138
BE						1.909
LS						

VIF values are reported only for structural paths directed toward endogenous constructs. Blank cells indicate that no direct path was specified. Construct abbreviations are defined in Table 1.

After confirming the absence of multicollinearity, the structural model was examined in terms of model approximation, explanatory power, predictive relevance, and standardized path coefficients. As a supplementary model approximation indicator for PLS-SEM, the standardized root mean square residual (SRMR) was 0.059, which is below the commonly suggested threshold of 0.08 and suggests adequate model approximation [48]. The significance of all structural paths was assessed using the PLS bootstrapping procedure [48].

The structural model was evaluated by examining the *R*^2^ values of the two endogenous constructs. As shown in Table 7, the *R*^2^ values were 0.476 for behavioral engagement and 0.647 for learning satisfaction. These values indicate that the model explained a meaningful proportion of variance in both constructs, with a higher level of explained variance for learning satisfaction than for behavioral engagement.

**Table 7:** *Q*^2^ and *R*^2^ values.

Variables	*Q* ^2^	*R* ^2^
BE	0.458	0.476
LS	0.577	0.647

Construct abbreviations are defined in Table 1.

To further assess the model’s predictive relevance, *Q*^2^ values were examined using the blindfolding technique (Table 7). The *Q*^2^ value was 0.458 for behavioral engagement and 0.577 for learning satisfaction. Because *Q*^2^ values greater than 0 indicate predictive relevance [48], these results suggest that the model demonstrates predictive relevance for both endogenous constructs in YIEL contexts.

**Fig 2:**
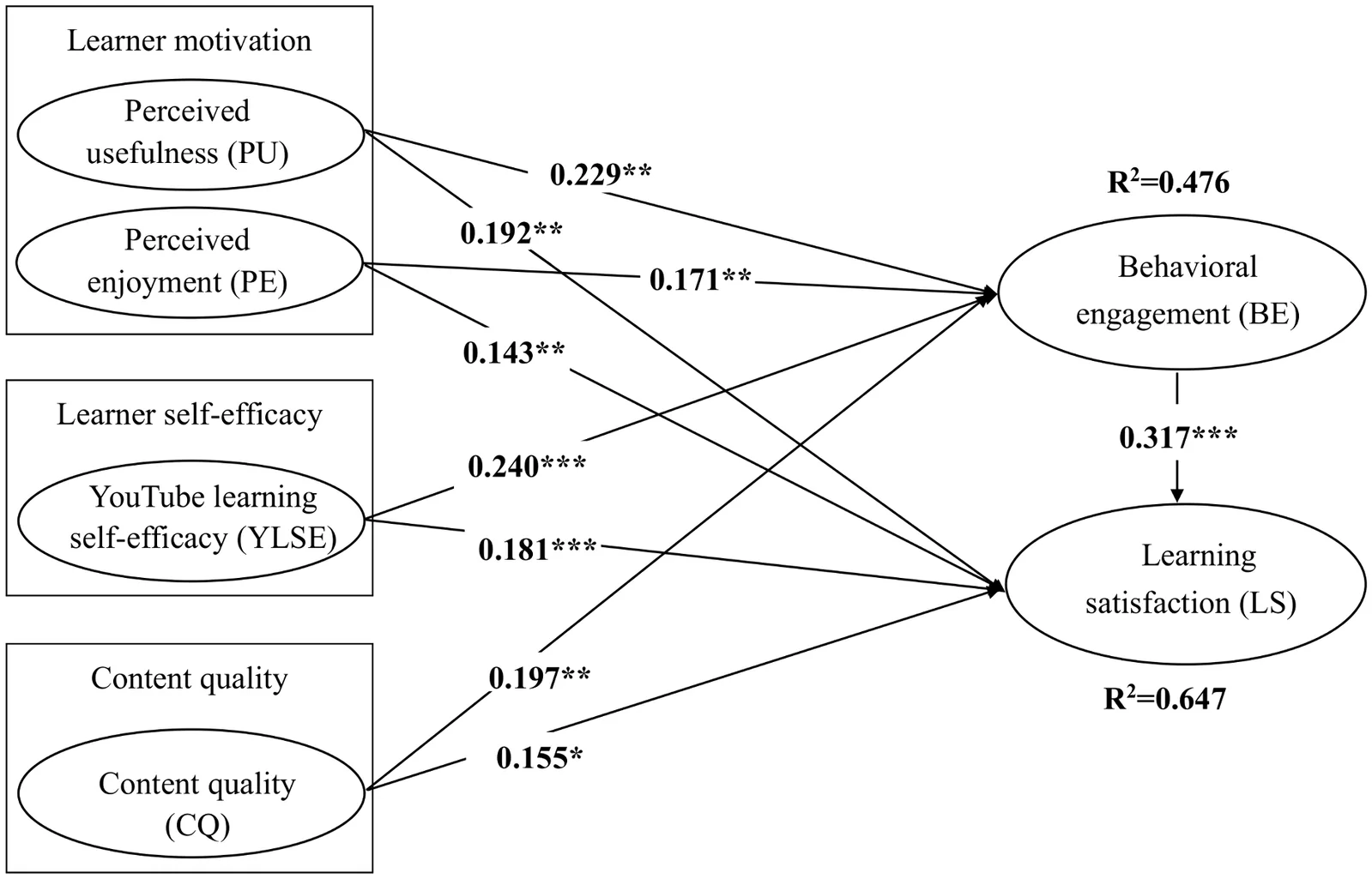
PLS analysis of the research model. *Note*: *p < 0.05; **p < 0.01; ***p < 0.001.

Table 8 summarizes the structural path results, and Fig 2 illustrates the overall PLS model. All hypothesized paths were positive and statistically significant; therefore, H1 through H9 were supported. Among the predictor constructs, YouTube learning self-efficacy had the largest coefficient for behavioral engagement (H5: *β* = 0.240, *p* < 0.001), whereas perceived usefulness had the largest direct coefficient for learning satisfaction (H2: *β* = 0.192, *p* = 0.002). Behavioral engagement was also positively associated with learning satisfaction (H9: *β* = 0.317, *p* < 0.001). Detailed coefficients, *t*-values, significance levels, and *f*² values are reported in Table 8.

**Table 8:** Results of structural model analysis.

Hyp.	Path	*β*	SE	*t*-value	*p*-value	*f* ^2^	Sig. level	Decision
H1	PU → BE	0.229	0.067	3.403	0.001	0.051	**	Supported
H2	PU → LS	0.192	0.063	3.068	0.002	0.052	**	Supported
H3	PE → BE	0.171	0.052	3.277	0.001	0.033	**	Supported
H4	PE → LS	0.143	0.050	2.880	0.004	0.034	**	Supported
H5	YLSE → BE	0.240	0.055	4.385	<0.001	0.061	***	Supported
H6	YLSE → LS	0.181	0.047	3.865	<0.001	0.048	***	Supported
H7	CQ → BE	0.197	0.068	2.883	0.004	0.036	**	Supported
H8	CQ → LS	0.155	0.065	2.397	0.017	0.032	*	Supported
H9	BE → LS	0.317	0.057	5.602	<0.001	0.149	***	Supported

*p < 0.05; **p < 0.01; ***p < 0.001. Construct abbreviations are defined in Table 1.

### Statistical mediation analysis

Behavioral engagement was further examined as a statistical mediator by testing the specific indirect associations between the four predictor constructs and learning satisfaction. The bootstrapping results showed that all indirect associations were significant (see Table 9). The indirect associations ranged from 0.054 to 0.076, and all *p*-values were below 0.01. These results provided statistical support for H10a–H10d, indicating that behavioral engagement statistically mediated the associations of perceived usefulness, perceived enjoyment, YouTube learning self-efficacy, and content quality with learning satisfaction.

**Table 9:** Results of statistical mediation analysis: specific indirect associations via behavioral engagement.

Hyp.	Indirect association	*β*	SD	*t*-value	*p*-value	Decision
H10a	PU → BE → LS	0.073	0.024	2.977	0.003	Supported
H10b	PE → BE → LS	0.054	0.021	2.598	0.009	Supported
H10c	YLSE → BE → LS	0.076	0.022	3.443	0.001	Supported
H10d	CQ → BE → LS	0.062	0.024	2.610	0.009	Supported

Construct abbreviations are defined in Table 1.

### Measurement invariance and multi-group analysis

Measurement invariance was assessed using the MICOM procedure in SmartPLS 4, following established guidelines for testing measurement invariance of composite models [51]. Compositional invariance was established for all constructs, and no significant differences were found in the means or variances across groups. These findings indicate full measurement invariance and allow for meaningful group comparisons.

Permutation-based multi-group analysis (MGA) was then conducted to examine group differences in structural path coefficients, with the results reported in Table 10 [51]. No significant differences were found in the structural path coefficients between male and female groups. For academic discipline, students from the College of Science and Technology were classified as the technology group, whereas students from the Colleges of Management, Education, and Humanities were classified as the non-technology group. Only the path from behavioral engagement to learning satisfaction differed significantly by academic discipline (*p* = 0.016), with a higher coefficient among non-technology students. No significant group differences were found for the remaining paths.

**Table 10:** Multi-group analysis (MGA) results.

Structural path	Gender (*p*-value)	Academic discipline (technology vs. non-technology) (*p*-value)
PU → BE	0.626	0.245
PU → LS	0.643	0.611
PE → BE	0.788	0.283
PE → LS	0.056	0.596
YLSE → BE	0.317	0.429
YLSE → LS	0.499	0.935
CQ → BE	0.794	0.052
CQ → LS	0.475	0.462
BE → LS	0.545	0.016*

Construct abbreviations are defined in Table 1. * *p* < 0.05.

## Discussion

This study examined how motivational factors, YouTube learning self-efficacy, and content quality are associated with behavioral engagement and learning satisfaction in YIEL. Perceived usefulness, perceived enjoyment, YouTube learning self-efficacy, and content quality were all positively associated with behavioral engagement and learning satisfaction in this learner-initiated and less regulated learning environment. Taken together, these findings characterize YIEL as a learner-managed context in which motivation, platform-specific confidence, and content evaluation are jointly related to behavioral engagement and learning satisfaction.

Perceived usefulness and perceived enjoyment were both positively associated with behavioral engagement and learning satisfaction, which is consistent with their proposed motivational relevance in YIEL. These findings are consistent with prior studies showing the relevance of perceived usefulness in blended and mobile-assisted English learning contexts [21,22]. They are also consistent with studies reporting associations of perceived enjoyment with engagement- and satisfaction-related outcomes in technology-supported language learning environments [20,24]. Together, these findings extend existing evidence to a more informal, learner-directed, and platform-mediated environment.

In YIEL contexts, perceived usefulness may be important because learners need to decide whether available videos are worth their time and effort. Learners who perceived YouTube as more useful also tended to report stronger behavioral engagement and greater learning satisfaction. From the perspective of Intrinsic Motivation Theory [14], one possible interpretation is that perceived enjoyment may be related to learners’ autonomy to choose engaging content and to the entertaining presentation styles common on YouTube. However, these possible sources of enjoyment were not directly examined in this study. The similar effect sizes of perceived usefulness and perceived enjoyment suggest that learners’ behavioral engagement and learning satisfaction in YIEL were associated with both perceived learning value and the enjoyment experienced during voluntary video-based learning. This may reflect the learner-directed nature of YIEL. In this context, instrumental value and affective enjoyment may represent distinct but complementary aspects of learners’ YIEL experiences.

Beyond motivational factors, YouTube learning self-efficacy was positively associated with both behavioral engagement and learning satisfaction. This finding aligns with previous research showing that online learning self-efficacy is positively associated with learner engagement [28] and is consistent with Social Cognitive Theory, which emphasizes task-specific efficacy beliefs. In less regulated environments such as YouTube, learners need to make independent decisions about how to locate, select, and use learning materials. Learners with stronger YouTube learning self-efficacy also tended to report greater behavioral engagement and more positive evaluations of their learning experience.

Content quality was positively associated with both behavioral engagement and learning satisfaction. This finding aligns with the Information Systems Success Model [31], although the associations of content quality with both outcomes were modest. In YIEL, learners engage with English learning videos without formal instructional filtering, and available materials may vary in accuracy, relevance, and pedagogical quality. When learners perceived videos as credible and relevant to their learning needs, they also tended to report greater behavioral engagement and learning satisfaction.

However, the effect sizes for content quality were among the smallest in the model (*f*² = 0.036 and 0.032), indicating a modest independent explanatory contribution. One possible interpretation concerns YouTube’s algorithm-mediated content exposure. YouTube’s recommendation system draws on multiple user-, context-, and interaction-related signals, including viewing and search histories and prior interactions with videos [34]. In this context, learners’ behavioral engagement and learning satisfaction may reflect broader aspects of their video-based learning experience beyond perceived content quality alone, including perceived usefulness and perceived enjoyment. When these learner perceptions are considered simultaneously, content quality may therefore provide relatively limited additional explanatory power. However, because the present study did not directly measure recommendation exposure or content-selection processes, this interpretation remains tentative and warrants further investigation.

Behavioral engagement was positively associated with learning satisfaction. This result is consistent with prior research [38], which suggests that active participation is closely related to learning satisfaction in online learning contexts. In the learner-directed YIEL environment, behavioral engagement may be particularly relevant because learners must manage their own participation without formal guidance. Students who reported greater attention, effort, and time investment in selecting and following learning content also tended to report higher learning satisfaction.

The mediation analysis further showed that behavioral engagement statistically mediated the associations of the four constructs with learning satisfaction. Each construct had a direct association with learning satisfaction and a statistically significant specific indirect association involving behavioral engagement. In the less regulated and learner-initiated YIEL context, students with more positive perceptions, stronger platform-specific confidence, and more favorable content evaluations tended to report greater behavioral engagement. Higher behavioral engagement was also associated with higher learning satisfaction. Given the cross-sectional design, these indirect relationships should be interpreted as statistical associations rather than causal pathways.

Viewed across the full model, the findings suggest that no single construct was consistently dominant in its associations with behavioral engagement and learning satisfaction. Rather, perceived usefulness, perceived enjoyment, YouTube learning self-efficacy, and content quality were each associated with both outcomes. Behavioral engagement also statistically mediated the associations of these four constructs with learning satisfaction. Thus, learning satisfaction was associated both with these constructs and with learners’ reported behavioral engagement.

### Additional model assessment

To further interpret the model, this section considers construct distinctiveness, effect sizes, potential overfitting, and subgroup stability. These checks help clarify the explanatory value and limitations of the proposed model in the YIEL context.

Construct distinctiveness was first examined using the HTMT criterion. Although content quality, behavioral engagement, and learning satisfaction were closely related, the HTMT values remained within acceptable ranges (CQ–LS = 0.854; BE–LS = 0.884), and their 95% bootstrapped confidence intervals did not include 1.0. These results suggest that the constructs are related but not interchangeable. Content quality reflects evaluations of available materials, behavioral engagement captures effort and attention, and learning satisfaction represents learners’ overall evaluation of the learning experience.

The *f²* values were also considered when interpreting the practical relevance of the structural paths. The *f²* values were small, ranging from 0.032 to 0.149, which is not uncommon in behavioral and educational research where learning outcomes are often associated with multiple factors. Although individual effect sizes were modest, the model explained meaningful proportions of variance in behavioral engagement and learning satisfaction. This pattern suggests that the four constructs provide explanatory value when considered together, rather than indicating the dominance of a single factor. At the same time, the small individual effect sizes limit the practical weight that should be assigned to any single construct.

The pattern of significant paths was also considered in relation to potential overfitting. Although all hypothesized associations were statistically supported, the path coefficients and *f²* values varied across constructs. The results were therefore not characterized by uniformly strong or inflated effects. Accordingly, support for all hypotheses should not be interpreted as evidence that the model is theoretically complete or free from confirmation bias. Instead, the model’s explanatory power appears to reflect the combined associations of the four theoretically grounded constructs with behavioral engagement and learning satisfaction. Nevertheless, this pattern should be interpreted with caution until it is replicated across different samples, platforms, or learning contexts. Future studies could further address this concern through preregistered hypotheses and comparisons with theoretically plausible alternative models.

Group differences were also examined using multi-group analysis to assess subgroup stability. No significant differences were found across gender groups, suggesting that the model operated similarly for male and female learners. For academic discipline, only the path from behavioral engagement to learning satisfaction differed significantly. This path was stronger among non-technology students. One possible interpretation is that, in this sample, behavioral engagement may have been more strongly associated with learning satisfaction among students from non-technology disciplines. The reason for this subgroup difference cannot be determined from the present data and should be examined in future research.

These checks support the model’s usefulness for describing the observed associations in YIEL, but the results require cautious interpretation.

### Theoretical implications

YIEL differs from traditional classrooms and institutionally structured online learning environments in that it is more decentralized, learner-initiated, and partly shaped by platform mechanisms. For this reason, YIEL offers a useful setting for examining the associations among learner motivation, self-efficacy, content evaluation, engagement, and satisfaction outside formal instructional structures. The findings have four theoretical implications.

First, this study proposes an integrated framework for understanding behavioral engagement and learning satisfaction in YIEL. It brings together motivational constructs from the Technology Acceptance Model and Intrinsic Motivation Theory, YouTube learning self-efficacy from Social Cognitive Theory, and content quality from the Information Systems Success Model. The findings indicate that behavioral engagement and learning satisfaction in YIEL are associated with motivational factors, YouTube learning self-efficacy, and content quality rather than with any one construct alone. Together, these theories provide a basis for interpreting the associations of these constructs with behavioral engagement and learning satisfaction. This framework may be especially relevant in decentralized, algorithm-mediated, and less regulated learning contexts.

Second, this study clarifies the contextual relevance of content evaluation in YIEL. Although the associations of perceived content quality with behavioral engagement and learning satisfaction were modest, content evaluation remains a relevant aspect of learners’ experiences in an algorithm-mediated and user-generated content environment. In formal online learning environments, learning materials are often selected or structured by instructors or institutions. By contrast, YIEL requires learners to judge whether YouTube can support their English learning goals and whether available videos are relevant, reliable, and suitable for their needs. Perceived content quality therefore provides a useful conceptual lens for understanding how learners evaluate and select English learning videos in decentralized and less regulated digital learning environments.

Third, the findings highlight the theoretical importance of YouTube learning self-efficacy. This construct reflects learners’ confidence in using YouTube for learning purposes and is more specific than general online learning self-efficacy or computer self-efficacy. This distinction is important in YIEL because learners must not only operate the platform, but also search for videos, compare available materials, select suitable resources, and decide how to use what they find.

Fourth, behavioral engagement was associated with learning satisfaction and statistically mediated the associations of motivational factors, YouTube learning self-efficacy, and content quality with learning satisfaction. This finding identifies behavioral engagement as a relevant construct in YIEL, where learners actively select, watch, and use learning materials. Although this study focuses on YIEL, this statistical pattern may also be examined in other forms of informal second-language learning on video-sharing platforms.

### Practical implications

The findings have practical relevance for content creators, learners, and platform designers involved in YIEL. Because this study was cross-sectional**,** the following suggestions should be viewed as tentative. The findings do not show that changing any one factor will necessarily improve learning outcomes. The availability of English learning videos alone is unlikely to be sufficient. Practical support for YIEL should consider learners’ perceived usefulness, perceived enjoyment, perceived content quality, platform-specific confidence, and active behavioral engagement. These factors suggest that content design, learner strategies, and platform features should help learners evaluate, select, and remain actively involved with English learning videos. The following implications are organized around these three stakeholder groups.

For content creators, greater attention could be directed toward strengthening learners’ perceptions of usefulness and enjoyment. These perceptions showed relatively stronger associations with behavioral engagement and learning satisfaction than those observed for content quality. Creators could clearly communicate the learning purposes of their videos, connect the materials to learners’ practical language needs, and illustrate how the content can be applied in everyday contexts. They could also consider using engaging and learning-oriented design features, such as narrative framing, brief opportunities for reflection, and contextualized examples. Although these specific features were not directly examined in this study, they may help strengthen learners’ perceptions of enjoyment and usefulness.

Content quality also remains relevant, and creators should continue to ensure that learning materials are accurate, credible, clearly presented, and appropriate for learners’ needs. However, because content quality showed relatively small effect sizes, it appears to have limited standalone practical importance. Accordingly, content quality should be viewed as one component of the broader learning experience rather than as the primary focus of content design.

For learners, the findings suggest the value of strengthening skills for locating, assessing, and applying English learning videos. These skills may help learners navigate a wide range of user-generated materials with greater confidence. Learners may consider explicitly connecting YouTube-based learning activities to their personal learning goals and adopting intentional learning behaviors, such as taking notes, reviewing key segments, returning to useful videos, and applying newly learned expressions.

For platform designers, the findings point to opportunities to improve system-level features that support informal English learning on YouTube. Recommendation systems could better support learners by allowing users to specify learning goals or proficiency levels and receive more relevant video suggestions. Learning-support tools, such as goal-based playlists, bookmarking functions linked to review reminders, progress tracking, and personalized review suggestions, may help learners manage their learning process. In addition, simple interactive or feedback-oriented features, such as reflection prompts or brief self-check activities, may make the learning process more enjoyable. Although these features do not directly control the quality of user-generated materials, they may help learners make better use of available content and manage their informal learning process more effectively.

Overall, the observed associations point to practical considerations involving both the design of English learning videos and the ways learners evaluate, select, and engage with them. Content creators may consider refining content presentation, learners may consider strengthening self-directed learning strategies, and platform designers may consider developing features designed to help learners manage and sustain engagement. The value of these proposed directions should be evaluated in future longitudinal or intervention-based research.

### Limitations and future research

The findings should be interpreted in light of several limitations. First, the sample consisted of undergraduate students from a single public university in central Taiwan and was obtained through convenience sampling. This may limit the generalizability of the findings to other EFL populations, institutional contexts, and cultural or national contexts. Because students’ informal English learning behaviors may also be shaped by the learning culture and digital learning practices of their institution, the findings should be interpreted with this institutional context in mind. Future studies could include participants from multiple institutions, regions, or educational levels to enhance generalizability.

Second, participants were recruited through a university-affiliated Facebook community. This approach may over-represent students who are more digitally active or more familiar with social media platforms. Future studies could combine online and offline recruitment channels to obtain a more diverse sample.

Third, the study used a cross-sectional design, which limits the ability to establish causal relationships or confirm the temporal ordering of the proposed associations. Accordingly, the findings should be interpreted as associations rather than causal effects. Longitudinal, experimental, or multi-wave designs would be useful for examining temporal dynamics and the direction of relationships.

Finally, the study relied on self-reported questionnaire data. Although diagnostic tests suggested that common method bias was not severe, common method variance cannot be entirely ruled out. Future research could incorporate learning logs, viewing records, platform-based behavioral data, or qualitative interviews to better capture learners’ actual engagement and reduce potential method-related bias.

## Conclusion

This study examined behavioral engagement and learning satisfaction in YIEL. The results indicate that perceived usefulness, perceived enjoyment, YouTube learning self-efficacy, and content quality are positively related to both outcomes. These findings suggest that YIEL can be understood as a learning context in which motivation, platform-specific confidence, and content evaluations are jointly associated with behavioral engagement and learning satisfaction.

Behavioral engagement also represented an important construct in the model. It was positively associated with learning satisfaction and statistically mediated the associations of these four constructs with learning satisfaction.

Overall, this study provides a clearer basis for understanding and supporting open, self-directed, video-based English learning. Satisfying YIEL experiences appear to involve not only perceptions of usefulness, enjoyment, and content quality, but also learners’ confidence and active behavioral engagement.

## Supporting information

S1 Appendix Questionnaire items. (PDF)
