# Supplementary material for: Factors associated with behavioral engagement and learning satisfaction in YouTube-based informal English learning among Taiwanese undergraduate students
Source: PLoS One. 2026 Aug 28;21(8):e0357471. doi: 10.1371/journal.pone.0357471 (PMC13524258; doi:10.1371/journal.pone.0357471)
Supplement: Supplementary file 1 [file pone.0357471.s001.pdf]

Summary of measurement items

Perceived usefulness

1. Using YouTube improves my English learning performance.
2. Using YouTube helps me learn English more efficiently.
3. Using YouTube enhances the effectiveness of my English learning.
4. Overall, YouTube is useful for my English learning.

Perceived enjoyment

1. I find using YouTube videos to learn English enjoyable.
2. The actual process of using YouTube videos for learning English is pleasant.
3. I have fun learning English through YouTube videos.

Content quality

1. The English learning videos I watch on YouTube are up-to-date.
2. The English learning videos I watch on YouTube are relevant to my English learning needs.
3. The English learning videos I watch on YouTube are reliable sources of information for English learning.
4. The English learning videos I watch on YouTube provide accurate information.

YouTube learning self-efficacy

1. I feel confident using YouTube's search function to find English learning videos relevant to my learning.
2. I feel confident navigating YouTube to locate relevant English learning videos efficiently.
3. I feel confident selecting and using English learning videos on YouTube effectively for learning purposes.
4. I feel confident independently exploring YouTube to discover new English learning videos.

Behavioral engagement

1. I watch and listen very carefully when learning English on YouTube.
2. I pay attention when learning English on YouTube.
3. I put a lot of effort into learning English through YouTube.
4. I work as hard as I can when using YouTube to learn English.

Learning satisfaction

1. Using YouTube to learn English was a wise choice.
2. I am happy with my experience of using YouTube to learn English.
3. Overall, I am satisfied with using YouTube to learn English.
